# Cellular and Molecular Mechanisms of Toxic Liver Fibrosis in Rats Depending on the Stages of Its Development

**DOI:** 10.17691/stm2023.15.4.05

**Published:** 2023-07-28

**Authors:** E.I. Lebedeva, A.T. Shchastniy, A.S. Babenka

**Affiliations:** Associate Professor, Department of Histology, Cytology and Embryology; Vitebsk State Order of Peoples’ Friendship Medical University, 27 Frunze Avenue, Vitebsk, 210009, the Republic of Belarus;; Professor, Head of the Department of Hospital Surgery with the Course of the Fetoplacental Complex and Placental Complex; Vitebsk State Order of Peoples’ Friendship Medical University, 27 Frunze Avenue, Vitebsk, 210009, the Republic of Belarus;; Associate Professor, Department of Bioorganic Chemistry; Belarusian State Medical University, 83 Dzerzhinsky Avenue, Minsk, 220116, the Republic of Belarus

**Keywords:** rat liver fibrogenesis, toxic fibrosis, mRNA expression, immunohistochemistry, Ishak’s scale

## Abstract

**The aim** is to study the cellular and molecular features of toxic liver fibrosis in rats and its dependence on development stages of this pathological condition.

**Materials and Methods:**

Liver fibrogenesis in male Wistar rats was induced with the thioacetamide solution by introducing into the stomach with a probe at a dose of 200 mg/kg of animal body weight 2 times per week. The process dynamics was studied at 5 time points (control, week 3, week 5, week 7, and week 9). The mRNA levels of *tweak*, *fn14*, *ang*, *vegfa*, *cxcl12*, and *mmp-9* genes in liver were detected by real-time polymerase chain reaction. Immunohistochemical study was performed on paraffin sections. The CD31, CD34, CK19, α-SMA, FAP, CD68, CD206, CX3CR1, and CD45 cells were used as markers. Fibrosis degree was determined in histological sections, stained in line with the Mallory technique, according to the Ishak’s semi-quantitative scale.

**Results:**

Two simultaneously existing morphologically heterogeneous populations of myofibroblasts expressing different types of markers (FAP, α-SMA) were identified in rat liver. Prior to the onset of transformation of fibrosis into cirrhosis (F1–F4, weeks 3–7), FAP^+^ and SMA^+^ cells were localized in different places on histological specimens. All stages of liver fibrosis development were accompanied by an increase in the number (p=0.0000), a change in the phenotypic structure and functional properties of macrophages. The CK19^+^ cells of the portal areas differentiated into cholangiocytes that formed interlobular bile ducts and ductules, as well as hepatocytes that formed rudiments of new hepatic microlobules. Pathological venous angiogenesis and heterogeneity of endotheliocytes of the intrahepatic vascular bed were detected. Two options for changes in mRNA expression of the selected genes were identified. The level of the *fn14* and *mmp-9* mRNAs at all stages of fibrosis was higher (p=0.0000) than in control rats. For *tweak*, *ang*, *vegfa*, and *cxcl12* mRNAs, the situation was the opposite — the level of genes decreased (p=0.0000). There were strong and moderate correlations between the studied target genes (p<0.05).

**Conclusion:**

It was established that the stages of toxic fibrosis had morphological and molecular genetic features. The FAP^+^ cells make the main contribution to development of portal and initial stage of bridging fibrosis. The stellate macrophages and infiltrating monocytes/ macrophages can potentially be used for development of new therapeutic strategies for liver pathology treatment. One should take into account the features of the markers’ expression by endothelial cells during the study of the intrahepatic vascular bed. Joint study of genes is a necessary *ad-hoc* parameter in fundamental and preclinical research.

## Introduction

Each year, approximately 2 million people in the world die from chronic liver diseases of various etiologies [1, 2]. By present, a lot of information has been accumulated on the cellular and molecular mechanisms leading to liver fibrosis and cirrhosis, potential strategies for treatment of these pathological processes have been proposed, but there is still no effective antifibrotic therapy provided [3-9]. The study of cellular and molecular patterns requires consideration of a significant number of factors at various levels of organization together with the analysis of genetic information, firstly, at the level of proteins, mRNA and DNA, and secondly, at the level of transcription and translation regulation processes involving short and long non-coding RNA, as well as processes using modification of the mRNA and DNA structure [7, 10].

Morphologically, liver fibrosis is manifested by a damage of the parenchyma lamellar structure, activation and transdifferentiation (changes in the phenotypic profile) of a number of cells, development of inflammation, pathological angiogenesis, and proliferation of fibrous connective tissue [8, 11–14]. Here, it seems logical to conduct research aimed at studying potential cells and target genes responsible for the mentioned pathological processes at the tissue, cellular, and molecular levels. However, due to a number of reasons (technical, financial, ethical, etc.), most authors do not thoroughly consider fibrogenesis in detail when studying the fibrosis cellular and molecular mechanisms, as well as do not take into account its key points, such as initiation and transition from the stage of fibrosis to cirrhosis. As a result, a significant part of information is lost [2, 11, 12].

In this study, the choice of cell populations and target genes was associated with the stages of fibrosis prior to its transition to cirrhosis. The authors considered such parameters as availability of inflammation, pathological angiogenesis and growth of fibrous connective tissue. Based on information from literature, 6 target genes were selected: *tweak* (*tnfsf12*), *fn14* (*tnfrsf12a*), *ang*, *vegfa*, *cxcl12* (*sdf*), and *mmp-9*, which were involved in molecular cascades associated with the specified parameters [15-20]. It is still unknown whether the products of these genes are independently involved in the processes of initiation and development of fibrosis, within the framework of the corresponding signaling pathways, or are closely interconnected to implement their functions and are elements of a complex process.

Fibrosis progresses and resolves with participation of parenchymal and non-parenchymal cells of the liver and other cells infiltrating the organ. There are controversial points of view to functions of these cells, changes in their number, phenotype, as well as localization of the fibrogenic cell population and various macrophage subpopulations (tissue stellate macrophages: M1, M2a, M2b, M2c and infiltrating macrophages, as well as the fibrogenic cell population) [21-27]. It is likely that the use of different experimental models and research methods determines the differences in interpretation of the results received.

Based on the above, one can assume that initiation and development of liver fibrosis of toxic etiology are accompanied by changes in the expression level of the selected molecular targets, as well as a change in the phenotypic profile of cells.

**The aim** is to study cellular and molecular features of toxic liver fibrosis in rats and its dependence on the stages of this pathological condition development.

## Materials and Methods

In this work, international terms on cytology and histology were used [28], and the terms adopted in the Guidelines for Nomenclature of Genes, Genetic Markers, Alleles, and Mutations in Mouse and Rat (http://www.informatics.jax.org/mgihome/nomen/gene.shtml) were used to describe target genes.

### Liver cells

For this study, fat-accumulating cells (liver stellate cells, lipocytes, perisinusoidal cells, Ito cells, pericytes, stellate cells) and portal fibroblasts were chosen as the main sources of intercellular substance; three macrophages subpopulations (tissue, of bone marrow origin, activated by an alternative anti-inflammatory M2 phenotype); bipotent liver stem cells and endotheliocytes.

### Experimental model

The authors used male Wistar rats with a weight of 190–210 g. The study was approved by the Commission on Bioethics and Humane Treatment of Laboratory Animals of the Vitebsk State Order of Peoples’ Friendship Medical University (Protocol No.6 dated 03.01.2019; Belarus). The study was conducted in accordance with the ethical principles of the European Convention for the Protection of Vertebrate Animals used for Experimental and Other Scientific Purposes (Strasbourg, 2006). The animals were kept in plastic-and-metal cages, 6 animals in each cage; the rats lived under natural light and had free access to food and water. The temperature in the vivarium was maintained at a level of 21–23°C, and the air humidity was approximately 50%.

Liver fibrosis was modeled by chronic intoxication with thioacetamide (TAA; Acros Organics, Belgium). In laboratory animals, TAA causes liver damage with morphological characteristics similar to those that people with liver fibrosis and cirrhosis have [29]. A freshly prepared TAA solution was administered intragastrically through a feeding tube at a dose of 200 mg/kg of body weight 2 times a week. The animals were randomized into 4 groups (n=12 per group) depending on the duration of TAA impact: 3 weeks (group 1), 5 weeks (group 2), 7 weeks (group 3), 9 weeks (group 4). Rats from the control group (n=12) received same volume of water without TAA intragastrically using a feeding tube.

### Histological, immunohistochemical, and morphometric studies

After guillotine decapitation under a short-term ether anesthesia, samples of 5–10 mm in diameter were taken from the large left lobe of the rat liver and placed in a 10% solution of neutral formalin (Biovitrum, Russia) in phosphate buffer and fixed for 24 h. One block for each staining method was sampled from each animal, and then blocks were cut using an HM340E microtome (MICROM Laborgeräte GmbH, Germany) into an average of 3–4 sections of 4-μm thickness and placed on glass slides. For plain histological preparations, liver sections were stained with hematoxylin and eosin, and in order to identify connective tissue, they were stained in line with the Mallory technique using the HMS70 staining machine (Thermo Fisher Scientific, Germany). Immunohistochemical study was performed on paraffin sections [30]. The list of markers is demonstrated in Table 1.

**Table 1:** List of markers used in the study

Marker name	Cell markers	Reference No.	Dilution
Monoclonal mouse antibodies CD31	Endothelial cells	E-AB-70173	1:500
Polyclonal mouse antibodies CD34	Mesenchymal stem/endothelial progenitor/endothelial cells	E-AB-60105	1:100
Monoclonal mouse antibodies α-SMA	Activated fat cells	E-AB-22155	1:1000
Polyclonal rabbit antibodies FAP	Activated portal fibroblasts	E-AB-32870	1:100
Monoclonal mouse antibody CD68	Tissue macrophages	E-AB-22013	1:200
Polyclonal rabbit antibody CD206	Functional state of tissue macrophages	E-AB-70178	1:500
Polyclonal rabbit antibodies CX3CR1	Macrophages of bone marrow origin	E-AB-33382	1:100
Monoclonal mouse antibodies CK19	Biliary stem liver cells	E-AB-70231	1:1000
Polyclonal rabbit antibodies CD45	Hematopoietic stem cells	E-AB-16319	1:200

The authors used antibodies manufactured by Wuhan Elabscience Biotechnology Co., Ltd (China), 2-step plus Poly-HRP Anti Rabbit/Mouse IgG Detection System/ with DAB Solution kit; Retrieve-All Antigen (Unmasking System Basic) and antibody dilution buffer (BioLegend Inc, USA); Twin-20 (Glentham Life Sciences, UK); PBS (Melford, UK). Better orientation in the preparation and correct identification of cells containing the studied antigen were achieved by counterstaining the sections with Mayer’s hematoxylin for 1 min. In order to objectively interpret the results for each examined series (animal group), positive and negative controls were used. Immunohistochemical staining was assessed as positive only if no staining was seen in the negative control and, vice versa, as negative if staining in the positive control was absent.

Histological preparations were examined using the ImageScope Color and Olympus cellSens Standard software. The area of connective tissue and the area of expression of CD31, CD34, and CK19 markers were determined as a percentage of the image area, without taking into account the degree of marker expression [31]. The measurements were conducted by microphotography of random fields of view of preparations using the Olympus XC30 digital camera (Japan) based on the Olympus BX51 microscope (Japan). The number of α-SMA+, FAP+, CD68+, CD206+, CX3CR1+, and CD45+ cells was counted in 12 fields of view of each histological section with a 40× lens magnification. The area of interlobular veins was measured in μm2. The degree of fibrosis was determined using the Ishak’s semi-quantitative scale [32].

### Assessment of the level of mRNA tweak, fn14, ang, vegfa, cxcl12, mmp-9

Liver pieces with a diameter of up to 5 mm were placed in cryovials and then stored in liquid nitrogen until the start of the procedure to isolate total RNA. The total RNA fraction was determined using the ArtRNA MiniSpin kit (ArtBioTech, Belarus) in line with the manufacturer’s instructions. The cDNA synthesis was conducted using oligo(dT) primers and the ArtMMLV Total reagent kit (ArtBioTech, Belarus) according to the manufacturer’s instructions. For each reaction, 200 ng of the total RNA fraction was used. The selection of oligonucleotide primers and guides for real-time polymerase chain reaction (real-time PCR) was made using the free online application Primer3 v. 0.4.0 (https://bioinfo.ut.ee/primer3-0.4.0/). The list of the selected molecular targets, the reference gene, and the sequences of oligonucleotide primers are shown in Tables 2 and 3.

**Table 2:** Characteristic features of target genes and reference gene used in the study

Gene name	Status	Gene ID in the NCBI database	Reference sequence mRNA	Coded protein
*tweak*	Target	360548	NM_001001513.2	Superfamily member of the tumor necrosis factor 12
*fn14*	Target	302965	NM_181086.3	Superfamily member of the receptors of the tumor necrosis factor 12A
*ang*	Target	305843	NM_001006992.1	Angiogenin
*vegfa*	Target	83785	NM_031836.3	Vascular endothelial growth factor A
*cxcl12*	Target	24772	NM_001033883.1	CXC subfamily chemokine
*mmp-9*	Target	81687	NM_031055.2	Matrix proteinase 9
*hes1* *	Candidate to reference genes	29577	NM_024360.4	Transcription factor 1 of the bHLH family

* the *hes1* was used as a reference gene, as it showed a high level of expression stability within the preliminary experiments; it was originally considered as a target gene [33].

**Table 3:** Sequences of specific oligonucleotide primers and fluorescently labeled guides of the target genes and the reference gene

Gene name	Oligonucleotide sequence, 5’ → 3’	Modification 5’/3’
*tweak* (direct)	CCCATTATGAGGTTCATCCAC	
*tweak* (reverse)	TCTCTTCCCAGCCACTCACT	
*tweak* (guide)	GACAGGATGGAGCACAGGCA	FAM/BHQ1
*fn14* (direct)	GGATGCGCAGCAGCAC	
*fn14* (reverse)	CAAAACCAGGGCCAGACTAA	
*fn14* (guide)	CCTGCCCACTTCAGGATGCT	FAM/BHQ1
*ang* (direct)	TGCGAAAGTATGATGAGGAGAA	
*ang* (reverse)	TGTTGCCATGGATAAAGGTG	
*ang* (guide)	ACCTCGCCCTGCAAAGAGGT	FAM/BHQ1
*vegfa* (direct)	GCAGATCATGCGGATCAAA	
*vegfa* (reverse)	ATGCTGCAGGAAGCTCATCT	
*vegfa* (guide)	CCTCACCAAAGCCAGCACAT	FAM/BHQ1
*cxcl12* (direct)	CAGATTGTTGCAAGGCTGAA	
*cxcl12* (reverse)	TCCACTTTAATTTCGGGTCAA	
*cxcl12* (guide)	AAGCAACAACAGACAAGTGTGCA	FAM/BHQ1
*mmp-9* (direct)	CTACTCGAGCCGACGTCAC	
*mmp-9* (reverse)	AGAGTACTGCTTGCCCAGGA	
*mmp-9* (guide)	GATGTGCGTCTTCCCCTTCG	FAM/BHQ1
*hes1* (direct)	GAAAGATAGCTCCCGGCATT	
*hes1* (reverse)	CGGAGGTGCTTCACTGTCAT	
*hes1* (guide)	CCAAGCTGGAGAAGGCAGACA	FAM/BHQ1

The authors used the real-time PCR method with reagents manufactured by Primetech (Belarus). The final volume of the reaction mixture was 25 μl and contained all the necessary components in the following concentrations: 2 mM of magnesium chloride, 0.1 mM of the deoxynucleotide triphosphates mixture, 500 nM of oligonucleotides, including a guide for real-time PCR, and 1.25 units of thermostable Taq DNA polymerase with the appropriate buffer solution. Thermal cycling mode: +95°C for 2 min, then 40 cycles: +95°C for 5 s, +60°C for 45 s. Detection via the FAM channel was conducted after each cycle using the CFX96 touch device (Bio-Rad, USA). The efficiency of the reactions was determined using the standard curve method and series of dilutions, concentrated cDNA samples. For each sample of biological material, real-time PCR was performed in triplicate. In each experimental and control group, all 12 samples were analyzed separately for the highest reliability and account for intragroup variation, as well as phenotypic heterogeneity in the level of gene expression.

The standard curve method was applied to assess the effectiveness of reactions, as well as the absolute number of copies corresponding to mRNA. The relative level of mRNA expression was determined using the 2–ΔΔCt method. The effectiveness of all reactions varied by less than 5% and reached 94–97%.

### Statistical analysis

The results of quantitative measurements were assessed using the following software: Statistica 10.0 (StatSoft, Inc.), Microsoft Office Excel (Microsoft Corp.). For each sample, the normality of the frequency distribution of each feature was determined. The samples were not small (n=60>50); thus, the check was conducted within the Lilliefors test. Data were presented as arithmetic means (M) and their respective confidence intervals (95% CI), median, and 15th and 85th percentile values (Me [15%; 85%]). The level of statistical significance of differences in the studied characteristics in groups with a normal distribution was assessed using Student’s t-test; if the samples differed from the normal distribution, the Mann– Whitney U-test was used. Spearman’s nonparametric rank correlation and Pearson’s parametric correlation were used to identify the dependency and its strength between the studied characteristics. Differences were considered statistically significant at p<0.05.

## Results

### Pathomorphological characteristics of the rat liver during the experiment

**Figure 1.**
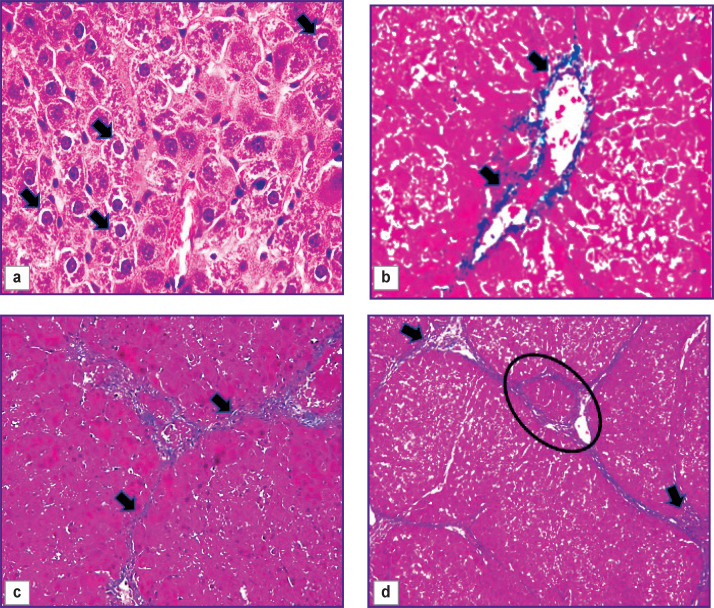
Pieces of rat liver 3 weeks (a, b), 7 weeks (c), and 9 weeks (d) after the start of the experiment: staining with hematoxylin and eosin, ×60 (a); staining in line with the Mallory technique, ×40 (b, c), ×20 (d); (a) perinuclear edema (*arrows*); (b) fibrous connective tissue in the portal area (*arrows*); (c) fibrous septa between portal areas (*arrows*); (d) false hepatic lobule (*oval frame*), angiogenesis in the portal areas (*arrows*)

Three weeks after the start of the experiment, the lamellar structure of the liver lobules was mostly preserved, but there were islands of 10–15 hepatocytes in necrobiosis in some areas of the parenchyma. Locally, single localized liver cells were with plasmolysis, karyolysis, and perinuclear edema development were seen (Figure 1 (a)). On the periphery of the classical hepatic lobules, the authors saw shapeless, swollen hepatocytes. The boundaries between them were indistinct and often did not differ. In most fields of vision, a thickening of the fibrous connective tissue around the portal areas and, less often, near the central veins (portal and centrilobular fibrosis, Figure 1 (b)) was seen. There were incomplete connective tissue septa formed at the portal areas, which ended blindly in the parenchyma. The degree of fibrosis was assessed as F1.

With further intoxication of rats (after 5 and 7 weeks), extensive areas of vacuolar dystrophy with pronounced plasmolysis and karyolysis of hepatocytes, as well as parenchymal edema were identified. Bridge-like connective tissue septa were formed, inducing convergence of the portal areas (bridging fibrosis, Figure 1 (c)). Along these fibrous septa, the authors saw hepatocytes exceeding normal cells in size by 1.5–2.0 times and having a large nucleus and dark oxyphilic cytoplasm. At that, areas with decomplexation of the lamellar structure of classical hepatic lobules and areas with unaffected parenchyma were seen. In separate areas, centrilobular and pericellular fibrosis was determined. This confirmed non-linearity of ongoing pathomorphological changes in the liver. The degree of fibrosis after 5 weeks was assessed as F2/F3, and after 7 weeks — as F3/F4.

After week 9, the process of nodular transformation of the parenchyma began near the triads with the formation of single false hepatic lobules (F4/F5, Figure 1 (d)). The authors did not detect increases in the areas of necrosis and necrobiosis of hepatocytes. At that, a significant proliferation of pathological connective tissue around the portal areas and formation of thick fibrous connective tissue septa along the periphery of the false hepatic lobules were seen.

The process of initiation and the initial stages of fibrosis development continued with a weak expression of cell infiltration (granular leukocytes, lymphocytes). At the F4/F5 stage, there was an increase in the number of infiltrate cells in the triads, fibrous septa, and parenchyma. Fatty degeneration was not diagnosed in the rat liver.

### Structural and functional derangements of the vascular bed of the rat liver

**Figure 2.**
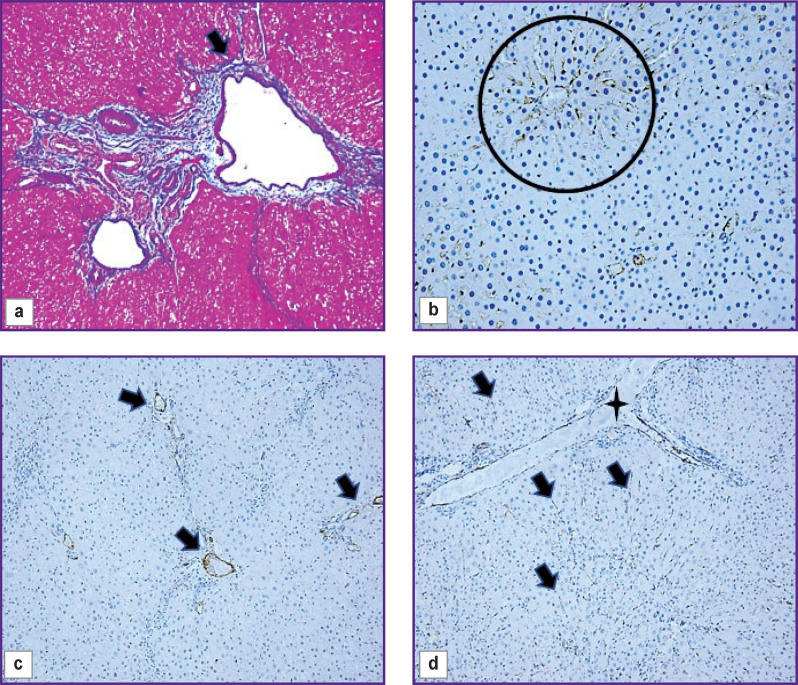
Pieces of rat liver of the control group (b), 5 weeks (a), and 3 weeks (c, d) after the start of the experiment: staining in line with the Mallory technique, ×20 (a); immunohistochemical staining for CD31, additional staining with Mayer’s hematoxylin, ×40 (b), ×20 (d); immunohistochemical staining for CD34, additional staining with Mayer’s hematoxylin, ×20 (c); (a) irregularly shaped interlobular vein (*arrow*); (b) CD31^+^ cells in the sinusoids of the central area of the classical hepatic lobule (*round frame*); (c) CD34^+^ cells in interlobular veins (*arrows*); (d) CD31^+^ cells in sinusoids (*arrows*) and interlobular vein (*star*)

At all stages of the experiment, an increase in the area of the interlobular veins (p=0.000) compared to the control group was seen. The veins were often gigantic and irregular in shape with many lacunae (Figure 2 (a)). Pronounced angiogenesis was observed in fibrous septa and portal areas (see Figure 1 (d)). It was manifested by formation of many venules and small veins. Endotheliocytes in venous vessels looked like vertical cells with uneven apical surface. The sludge phenomenon of erythrocytes was seen in the vessels. Almost no changes were identified in the interlobular arteries. In loci of significant expansion of the sinusoidal capillaries, Disse spaces were visualized. The central veins were practically not detected on sections.

**Figure 3.**
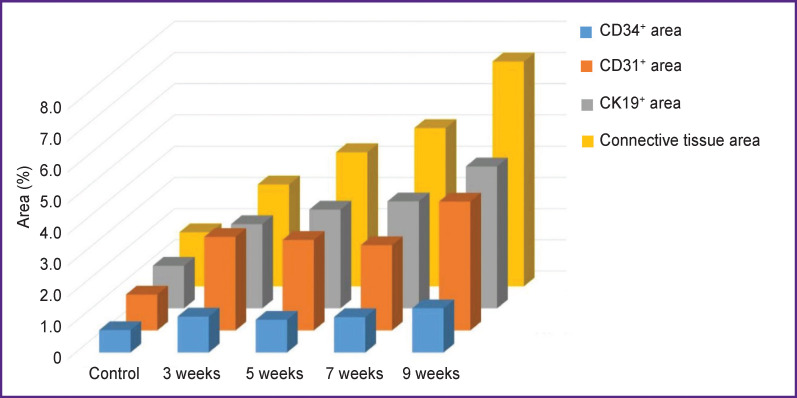
Change in the area of CD31^+^, CD34^+^, CK19^+^ cells with the development of fibrous connective tissue

In the liver of control and all experimental rats, endothelial cells of interlobular arteries, interlobular, central, and sublobular veins expressed CD34 and CD31 markers. In sinusoidal capillaries, the CD31 marker showed distribution by areas (Figure 2 (b)). In the central and periportal areas of the classical lobule, CD31+ cells with more intense staining were visualized; in the intermediate part, the authors saw a decrease in expression, in some places with a complete absence of an immunohistochemical reaction. The progression of fibrogenesis was accompanied by an increase in the area of CD31+ cells (p=0.0000) in sinusoidal capillaries (Figure 2 (d); Figure 3). CD34+ cells were not detected in the sinusoidal capillaries of the control and experimental rats; these cells were seen only in the interlobular veins (Figure 2 (c)).

Histological preparations revealed lymphatic vessels of large diameter, which clearly indicated development of lymphostasis against the background of progressive congestive processes. Dilated and deformed lymphatic vessels did not express CD34 and CD31 markers.

### Morphological and functional changes in FAP^+^, α-SMA^+^, and CD45^+^ cells

**Figure 4.**
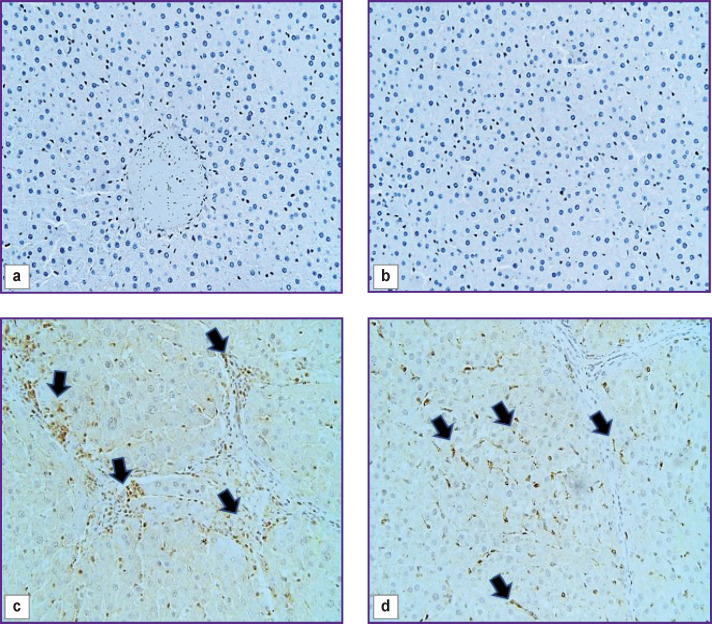
Pieces of the rat liver of the control group (a, b) and 9 weeks (c, d) after the start of the experiment: immunohistochemical staining for FAP, additional staining with Mayer’s hematoxylin, ×40 (a, c); immunohistochemical staining for α-SMA, staining with Mayer’s hematoxylin, ×40 (b, d); (a) FAP^+^ cells are missing; (b) α-SMA^+^ cells are missing; (c) FAP^+^ cells in fibrous septa (*arrows*); (d) α-SMA^+^ cells in sinusoids (*arrows*)

**Figure 5.**
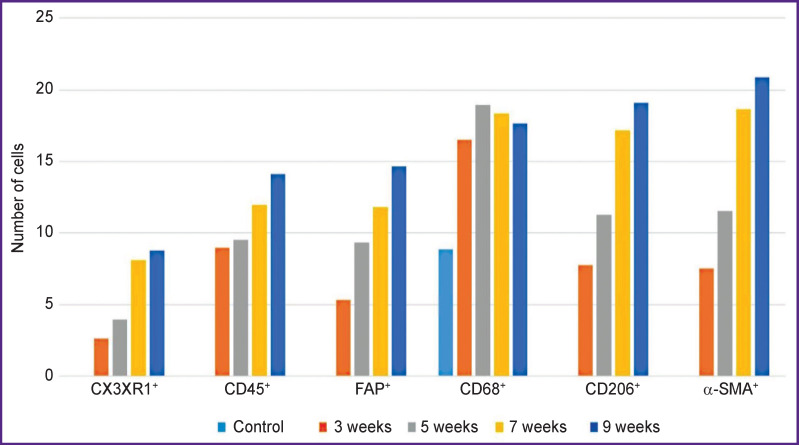
Change in the number of CD68^+^, CD206^+^, CX3CR1^+^, CD45^+^, FAP^+^, and α-SMA^+^ cells with the fibrous connective tissue growth

There were no cells expressing the FAP marker in the liver of control rats (Figure 4 (a)). In c-SMA sinusoidal capillaries, α-SMA+ cells were not seen (Figure 4 (b)), but in some cases these cells were detected in the walls of interlobular arteries, interlobular and sublobular veins. At the F1 stage, FAP+ and α-SMA+ cells were identified. With F2/ F3 bridging fibrosis, the number of FAP+ cells increased (p=0.0213), whereas α-SMA+ cells number did not change (p=0.3075) compared with the period of 3 weeks. At all subsequent stages of fibrosis, an increase in the number of both FAP+ cells (p=0.0000) and α-SMA+ cells (p=0.0000) was identified compared to the period of 3 weeks (Figure 5).

Two morphologically heterogeneous populations of myofibroblasts expressing different types of markers had a distinctive localization in the initial stages of the study. Before the beginning of transformation of fibrosis into F4/F5 cirrhosis, α-SMA+ cells were detected in sinusoidal capillaries (Figure 4 (d)) and necrosis loci, and at the end of the experiment they were also seen in the connective tissue septa. FAP+ cells were located around the interlobular vessels and near the interlobular bile ducts; starting from the F2/F3 stage, they were detected in fibrous trabeculae (Figure 4 (c)) and, less frequently, in sinusoidal capillaries.

The authors identified growth of CD45+cells, however, as fibrosis progressed, its rate slowed down (see Figure 5). In the liver of experimental rats, CD45+ cells were localized in triads and septa of fibrous connective tissue.

### Changes in macrophage subpopulations in rat liver

**Figure 6.**
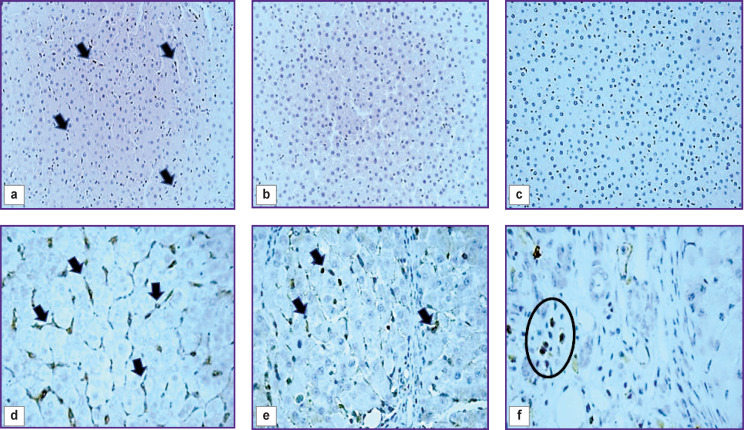
Pieces of the rat liver of the control group (a, b, c), 5 weeks (d) and 7 weeks (e, f) after the start of the experiment: immunohistochemical staining for CD68, staining with Mayer’s hematoxylin, ×40 (a), ×60 (d); immunohistochemical staining for CD206, additional staining with Mayer’s hematoxylin, ×40 (b); ×60 (e); immunohistochemical staining for CX3CR1, additional staining with Mayer’s hematoxylin, ×40 (c), ×60 (f); (a, d) CD68^+^ cells in sinusoids (*arrows*); (b) CD206^+^ cells are missing; (c) CX3CR1^+^ cells are missing; (e) CD206^+^ cells in sinusoids (*arrows*); (f) a group of CX3CR1^+^ cells in the portal area (*oval frame*)

In the control rats’ liver, wing-shaped CD68+ cells were seen predominantly in sinusoidal capillaries (Figure 6 (a)). An insignificant number of these cells were detected near the interlobular vessels and central veins. Cells expressing CD206 and CX3CR1 were extremely rare (Figure 6 (b) and (c)).

At all stages of fibrosis, the number of CD68+, CD206+, and CX3CR1+ cells (p=0.0000 in all cases) exceeded the control level (see Figure 5). One subpopulation of CD68+ cells was wing-shaped and localized predominantly in sinusoidal capillaries. More often these cells were placed one after another, forming chains (Figure 6 (d)), but sometimes they were localized side by side in two or three cells, contacting each other. The second subpopulation of CD68+ cells was round in shape and was found in different places of the histological section: around the blood vessels and interlobular bile ducts of the triads, in the loci of hepatocyte necrosis; cells surrounded accumulations of brown pigment, forming a ring; sometimes they accumulated in groups around single or groups of giant hepatocytes and liver cells containing brown pigment in the cytoplasm. Round-to-elongated CD206+ cells were mainly detected in sinusoidal capillaries in the form of chains at all stages of the experiment (Figure 6 (e)). At the beginning of the study, rounded CX3CR1+ cells were localized mainly in portal areas, whereas at subsequent stages they were seen in fibrous septa (Figure 6 (f)). There were areas of liver sections with a distinct migration of CX3CR1+ cells from the lumen of the vessel into the parenchyma. This proves their bone-marrow origin.

### Morphological and functional assessment of CK19^+^ cells

**Figure 7.**
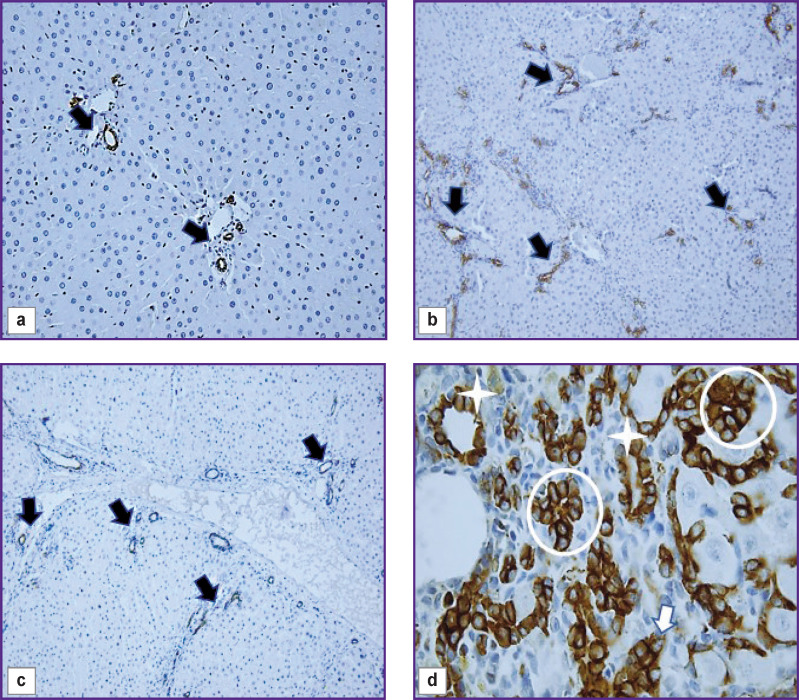
Pieces of the rat liver of the control group (a), 3 weeks (b), and 5 weeks (c, d) after the start of the experiment: immunohistochemical staining for CK19, staining with Mayer’s hematoxylin, ×40 (a), ×20 (b, c), ×100 (d); (a, b, c) interlobular bile ducts (*arrows*); (d) accumulation of CK19^+^ cells without a lumen (*arrow*), accumulation of CK19^+^ cells with a developing lumen (*round frame*), accumulation of CK19^+^ cells with a formed lumen (*star*)

The cytokeratin CK19 biliary marker was intensely expressed in cholangiocytes of the ductules and ducts of the liver of control rats (Figure 7 (a)). The cells of the epithelium of the interlobular ducts had a cubic shape, light and large nuclei with hyperbasophilic nucleoli. Epithelial CK19+ cells of the bile ducts were closer to a flat shape with brown-stained cytoplasm. Liver fibrogenesis was accompanied by an increase in the number of interlobular bile ducts, ductules, and single CK19+ cells in triads and fibrous septa (see Figure 3; Figure 7 (b) and (c)). The authors observed stages of formation of interlobular bile ducts: from rounded rosette-like clusters of cells without a lumen and structures with a developing lumen to fully formed ducts (Figure 7 (d)).

### Expression of mRNA of the studied genes

**Figure 8.**
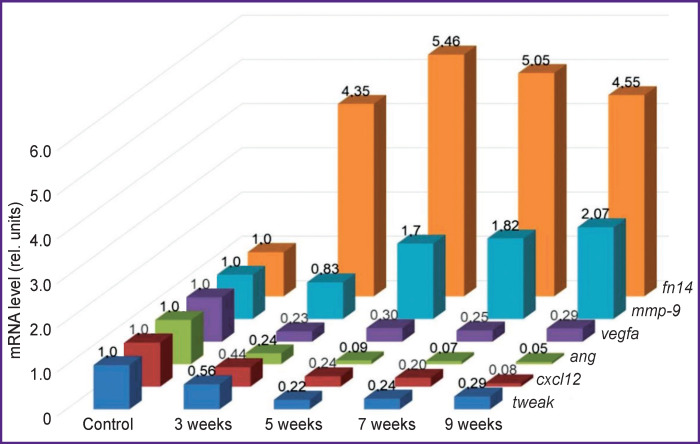
Changes in the mRNA expression level of the *tweak*, *fn14*, *ang*, *vegfa*, *cxcl12*, and *mmp-9* genes during the fibrous connective tissue growth

The selected target genes responded differently to fibrosis progression. The authors identified two ways of changing their expression (Figure 8). An increase in *fn14* and *mmp-9* mRNA was seen. The mRNA level of the *fn14* gene at all stages of fibrosis was higher (p=0.0000) than in the rats of the control group. At the F1 stage, *mmp-9* mRNA expression slightly decreased, and then it increased (p=0.0000) compared to the control group.

For the other targets, the situation was the opposite — the level of mRNA genes decreased. A drastic decrease was seen for the *ang* (p=0.0000) and *cxcl12* (p=0.0000) genes compared with the control group. The decrease in *vegfa* (p=0.0000) and *tweak* (p=0.0000) mRNA was not drastic and almost identical at stages F2/F3–F4/F5.

## Discussion

### Connective tissue

A thorough study of source of the fibrogenic cell population is of fundamental importance for development of antifibrotic drugs [21, 22]. In this study, the number of α-SMA+ cells increased linearly. The results obtained are consistent with the data by other authors [34, 35]. FAP+ cells at the F1 stage were located in portal areas next to CK19+ cells. It was established that CK19+ cells express profibrogenic factors (TGF-β, PDGF) and can activate portal fibroblasts [36]. Using histological preparations, the authors observed a directed growth of pathological connective tissue fibers with FAP+ cells from two portal areas through the liver parenchyma towards each other, which predetermines the path for fibrous tissue and formation of connective tissue bridges — bridging fibrosis. The beginning of the process of the parenchyma nodular transformation was near the portal areas.

It is well known that the main function of fibroblasts is the synthesis of intercellular substance [21, 37]. It is assumed that, after FAP+ cells receive a signal from the altered state of the niche (microenvironment), they are the first to synthesize the intercellular substance in the hepatic triads and, perhaps, regulate its secretion, participating in the formation of pathological septa (bridges). α-SMA+ cells act as myofibroblasts later. It is likely that the stage of activation and transdifferentiation of fat-accumulating cells is longer or the molecular signals that regulate the change in cell phenotype are induced with a delay or are inhibited until a certain point. The main site of localization of fat-accumulating cells is sinusoidal capillaries. At the initial stages, α-SMA+ cells are responsible for pericellular fibrosis and connective tissue synthesis in necrosis loci. One cannot exclude that the process of activation of portal fibroblasts and fat-accumulating cells is regulated by various mechanisms, which are yet to be studied.

The role of the FAP protein and its pharmacological inhibition as a potential therapy for liver fibrosis are not clearly examined. It is believed that the FAP protein stimulates inflammation, performs a profibrogenic function, and takes part in the regulation of energy and lipid metabolism [36, 37]. In this study, fatty degeneration was not detected, and a weak degree of cellular infiltration (granular leukocytes, lymphocytes) was seen up to the stage of F4/F5 fibrosis. Assumedly, the functions of the FAP protein are determined by the state of the niche (epigenetic mechanisms, gene activity, phenotypic cell profile, connective tissue density).

### Cells

The number, localization, phenotypic structure, and functional properties of macrophages change in the rat liver at different stages of TAA-induced fibrosis. According to some researchers, cells expressing the CD68 marker are resident (local) stellate macrophages and are characterized by plasticity, changing their phenotype in response to niche signals (classically activated M1 phenotype and alternatively activated M2 phenotype) [23, 24]. Some scientists consider the binary classification incompetent, assuming that there are several intermediate states of these cells’ differentiation [2, 38]. Using the immunohistochemical method, the authors revealed three morphologically different groups of stellate macrophages, differing in shape and localization. Apparently, a round-shaped subpopulation of CD68+ cells performs a predominantly phagocytic function, whereas a wing-shaped subpopulation of CD68+ cells replenishes the CD206+ cells population (an anti-inflammatory M2 phenotype of stellate macrophages), and they, in turn, support a weak degree of cell infiltration. As fibrosis progressed, the number of CD45+ cells decreased. At that, the population of CX3CR1+ cells increased. The results of this study are consistent with the data of other studies [2, 24]: in liver pathology, the monocyte formation in the red bone marrow increased, their migration and differentiation in macrophages accelerated. The authors do not report the exact localization and number of infiltrating macrophages, as well as they do not specify at what fibrosis stage they appear. In this work, CX3CR1+ cells were located in triads and fibrous septa, predominantly forming groups from 4 to 8, and were extremely rarely seen as single cells. These cells were found at the stage of portal fibrosis. This might indicate their participation in regulation of the functions of pathological septa polymorphic cells.

In many chronic liver diseases, proliferation of cholangiocytes (ductal or ductular reaction) is seen in hepatic triads. There is some disagreement regarding the role of this reaction [27]. Some scientists believe that ductular reaction cells are involved in liver regeneration by differentiating into hepatocytes [39]. Other authors establish a correlation between ductular response and liver fibrosis and suggest that it enhances fibrogenesis [40]. In this study, CK19+ cells of the portal areas differentiate into cholangiocytes, which form interlobular bile ducts and ductules, and hepatocytes, which form the rudiments of new hepatic microlobules.

### Intrahepatic vasculature

In the rat liver, there were no pronounced changes in the arterial system seen. One can assume that this is due to restructuring of the cells’ energy metabolism and the increased role of glycolysis in energy production. At that, the amount of energy and metabolites required to intensify the synthesis of extracellular matrix proteins increases. Demand in oxygen remains practically unchanged. An increase in portal blood flow is associated with widening of the interlobular veins’ diameter and pathological venous angiogenesis. The change in the shape and apical pole of endothelial cells in venous vessels can be characterized as compensation for the blood flow obstruction in the portal vein system due to development of edema and mechanical compression of veins in fibrosis.

The absence of the CD34 marker expression in the endotheliocytes of the sinusoidal capillaries of control and experimental rats indicates the morphological heterogeneity of the endothelial cells of the liver and is related to the vessels’ structure. The capillarization of sinusoids revealed in the present study is consistent with the other studies [5, 12, 13, 41]. This process probably precedes the activation of fat-accumulating cells and contributes to fibrosis development. An increase in the diameter of the lymphatic vessels clearly indicates the development of lymphostasis against the background of progressive stagnant processes.

### Target genes

**Figure 9.**
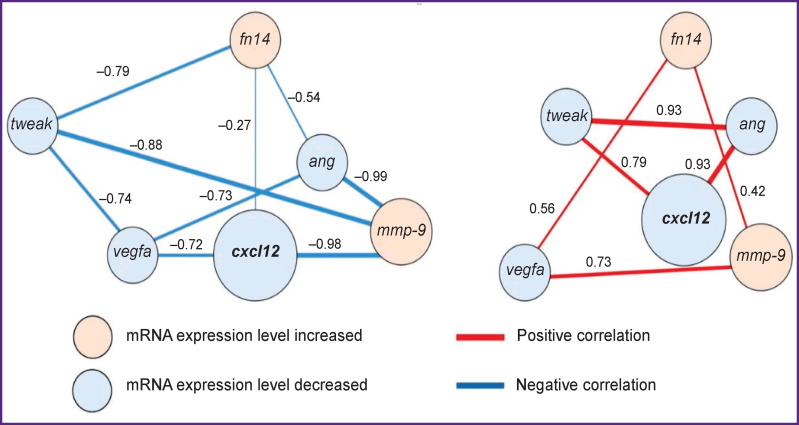
Correlations between the *tweak*, *fn14*, *ang*, *vegfa*, *cxcl12*, and *mmp-9* genes during the fibrous connective tissue growth

The authors revealed positive and negative strong or moderate correlations between the studied genes (*tweak*, *fn14*, *ang*, *vegfa*, *cxcl12*, and *mmp-9*) during the development of liver fibrosis (p<0.05, Figure 9).

The largest number of links was established for *cxcl12*. The CXCL12 protein is responsible for a series of important functions, including chemotaxis, cell migration and adhesion, proliferation, etc. [42]. The *ang* and *vegfa* products are involved in angiogenesis processes; however, at the mRNA level, they are linked to a much larger number of molecular processes [12, 13, 16, 17]. It is known that the MMP-9 protein plays a key role in the connective tissue renewal processes and controls its normal formation [15]. The Tweak/Fn14 complex is considered to promote accumulation of connective tissue in organs [18, 19, 20, 43].

Assumedly, the correlations established in this study for mRNA levels of the target genes may indicate their role in liver fibrogenesis. Joint related study of genes is a necessary *ad-hoc* parameter in fundamental and preclinical research.

**Figure 10.**
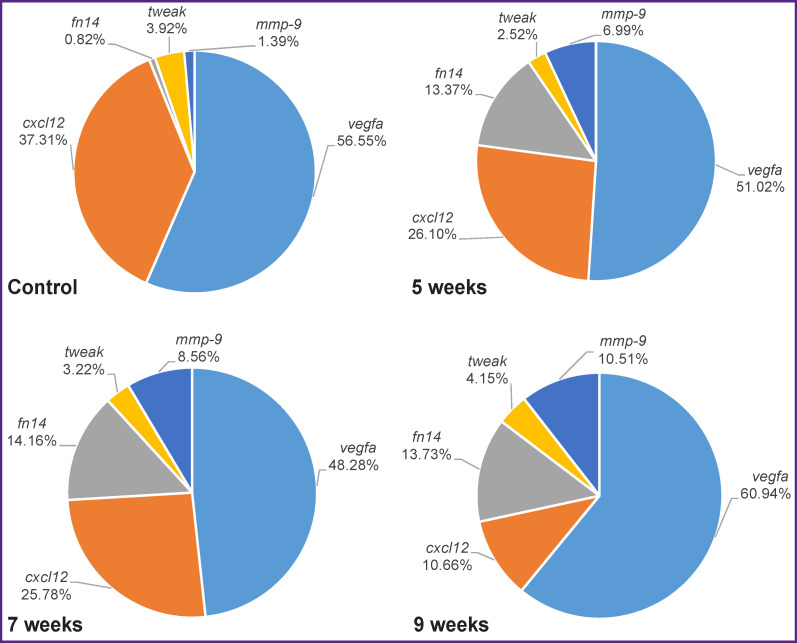
Percentage of the absolute number of mRNA copies of the *tweak*, *fn14*, *ang*, *vegfa*, *cxcl12*, and *mmp-9* genes during the fibrous connective tissue growth

It is interesting to note the presence of a strong correlation between genes, whose mRNA levels decreased relative to the control values. This can be explained by the fact that, despite the general trend, the decrease in the mRNA expression level occurred nonuniform for different genes. This process led to a change in the initial percentage relations of the absolute number of mRNA copies (Figure 10). For example, in the case of the *vegfa* gene, the percentage of its mRNA relative to other target genes averaged 54.2% and varied within 12.5% during the experiment. At that, the percentage of mRNA of the *cxcl12* gene decreased by 3.5 times (from 37.30 to 10.66%) also with a decrease in the relative level of the mRNA expression.

## Conclusion

Based on the study results, the authors established that the stages of toxic fibrosis have morphological and molecular genetic features. FAP+ cells are the major contributors to the development of portal and initial stage of bridging fibrosis. These cells can be considered as one of the myofibroblast populations in thioacetamide-induced liver fibrogenesis.

The progression of liver fibrosis is accompanied by a change in the number, localization, phenotypic structure, and functional properties of macrophages.

Pathological venous angiogenesis was identified. During the study of the intrahepatic vascular bed, one shall consider the specific features of the markers’ expression with endothelial cells.

Significant correlations (r=0.42–0.98; p<0.05) were detected between the target genes *tweak*, *fn14*, *ang*, *vegfa*, *cxcl12*, and *mmp-9*, confirming their role in development and initiation of liver fibrosis.
